# Respiratory Symptoms due to Occupational Exposure to Formaldehyde and MDF Dust in a MDF Furniture Factory in Eastern Thailand

**DOI:** 10.1155/2016/3705824

**Published:** 2016-12-14

**Authors:** Anamai Thetkathuek, Tanongsak Yingratanasuk, Wiwat Ekburanawat

**Affiliations:** ^1^Department of Industrial Hygiene and Safety, Faculty of Public Health, Burapha University, Chonburi 20131, Thailand; ^2^Occupational Medicine Center, Samitivej Sriracha Hospital, Chonburi 20110, Thailand

## Abstract

The study aimed to investigate factors associated with respiratory symptoms in workers in a medium-density fiberboard (MDF) furniture factory in Eastern Thailand. Data were collected from 439 employees exposed to formaldehyde and MDF dust using questionnaire and personal sampler (Institute of Occupational Medicine; IOM). The average concentration of formaldehyde from MDF dust was 2.62 ppm (SD 367), whereas the average concentration of MDF dust itself was 7.67 mg/m^3^ (SD 3.63). Atopic allergic history was a factor associated with respiratory irritation symptoms and allergic symptoms among the workers exposed to formaldehyde and were associated with respiratory irritation symptoms and allergic symptoms among those exposed to MDF dust. Exposure to MDF dust at high level (>5 mg/m^3^) was associated with respiratory irritation symptoms and allergic symptoms. Excluding allergic workers from the study population produced the same kind of results in the analysis as in all workers. The symptoms were associated with the high concentrations of formaldehyde and MDF dust in this factory. If the concentration of MDF dust was >5 mg/m^3^, the risk of irritation and allergic symptoms in the respiratory system increased. The respiratory health of the employees with atopic allergic history exposed to formaldehyde and MDF dust should be monitored closely.

## 1. Introduction

Medium-density fiberboard (MDF) is composed of the residues of wood production, such as hardwood sawdust (equivalent to 0–15%) and softwood sawdust (85–100%) mixed with wax, resin, or glue [1, 2] and combined with 8–18% urea–formaldehyde resin [3]. The board is then heat-pressurized to form shapes. It is therefore known as particle board [4]. The MDF furniture making process included tasks such as cutting, sawing, drilling holes, polishing, and gluing. Workers are at-risk of exposure to MDF dust and formaldehyde released from the work process and the storage of raw materials and the finished products. Formaldehyde has been classified by the International Agency for Research on Cancer (IARC) as Group 1 human carcinogen [5, 6].

The noncarcinogenic effects of MDF dust and formaldehyde include eye and skin irritation [2, 7, 8], dermatitis, [9] respiratory illnesses such as nasal inflammation [10], asthma [7, 11, 12], bronchitis [13], coughing, loud breathing, and wheezing [11, 12], and lower chest discomfort [8]. Compared to those not exposed to MDF products, their lung function, for example, forced vital capacity (FVC) and forced expiratory volume in 1 s (FEV_1_), may also be reduced [7, 14, 15].

Exposure to MDF dust and formaldehyde varied among employees from different working departments in the production process. As regards the type of wood, in terms of statistical significance, the amount of dust from MDF is greater than that from soft wood [16]. Apart from the type of wood and the size of the particles [17], the amount of dust also depends on the composition of the MDF, such as the connecting substances (e.g., glue). Concerning the efficiency of factory control, an appropriate ventilation system will reduce the concentration of MDF dust and formaldehyde, but the dust control systems in factories located in Southeast Asia are not usually very efficient [18], and so personal hazard preventions include the use of masks and so on [19].

Surveillance of the workplace environment can be determined by assessing the concentrations of formaldehyde and MDF dust. The standard concentration of formaldehyde set by the Occupational Safety and Health Administration (OSHA) is 0.75 ppm [20]. A study conducted by Vaizoğlu et al. [21] indicated that the concentration of formaldehyde at 100 furniture factories in Japan was 0.6  ppm (SD 0.3). However, the concentration of formaldehyde in the Southeast Asian region is usually higher than the permissible exposure limits (PELs) mentioned above.

Regarding the standard concentration of inhalable MDF dust set by American Conference of Governmental Industrial Hygienists (ACGIH) [22], it was determined that the dust of all softwoods and hardwoods except western red cedar should have a threshold limit value (TLV) of 1 mg/m^3^. The UK Health and Safety Executive (HSE) has suggested a standard scale for dust exposure (the workplace exposure limit) of 2.5 mg/m^3^ [23]. As regards the monitoring of employees' health, the respiratory health screening was achieved through lung function tests [24]. Questionnaires were also used in the preliminary screening for disorders, such as the questionnaire from the American Thoracic Society (ATS) [25].

Previous studies were concerned with work-related concentrations of formaldehyde and MDF dust, for example, exposure to respirable and inhalable dusts during the cutting and polishing of MDF and softwoods [16]. Health effects of exposure to MDF dust contributed to asthma and tracheitis after exposure to MDF dust [3, 8]. Impaired lung functions were associated with exposure to natural rubber-wood dust in furniture factory workers [26]. Respiratory system and skin effects were caused by exposure to the wood dust of the rubber tree* Hevea brasiliensis *[27]. However, there was no study on factors related to respiratory system disorders due to exposure to formaldehyde and MDF dust in the furniture factory. This study was conducted to provide information on preventive measures, and for health monitoring for the employees, with an aim to investigate factors related to respiratory symptoms among the MDF furniture factory workers in Eastern Thailand.

## 2. Materials and Methods

### 2.1. Study Design

This was a cross-sectional study conducted between January 2015 and August 2015. The study population consisted of employees at a MDF furniture factory in Chacheongsao Province, Thailand.

### 2.2. Study Population

The total number of employees at this factory was 535; however, 432 volunteered to participate in the study with informed consent. The number of the samples collected for formaldehyde and MDF dust exposure was calculated from the formula proposed by the American Industrial Hygiene Association, which determined the size of the samples to be used in the assessment of workers exposed to chemicals each day using the random sampling method in a homogeneous group. The appropriate number of samples was 6–10 samples [28].

In the furniture production process there are 10 working departments which cover similar exposure groups (SEG), namely, office, combination, edging, fitting, clearing, drilling, laminating, wrapping, packing, and line. This meant that there were 100 samples for the formaldehyde assessments and 60 for MDF dust exposure.

All employees selected to participate in the study volunteered willingly and without coercion, and consent forms were signed before participation. The study was approved by the Ethical Committee for Research in Humans of Burapha University, Thailand.

### 2.3. Data Collection

The data collection tools used in this study included questionnaires, and the equipment used to assess MDF dust and formaldehyde exposure are described below.

#### 2.3.1. Questionnaire

The researchers developed the questionnaire based on the American Thoracic Society Respiratory Symptom Questionnaire [25], adjusted to fit working conditions in the furniture factory. The subjects answered the questionnaire by themselves. The data collectors were occupational hygiene officers and public health academic staff who had been fully trained administering the questionnaire. The questionnaire consisted of four parts including general information, working history and environment, smoking and drinking history, and the history of respiratory disorders, which was divided into two groups of symptoms: irritation and allergic symptoms. Answers were marked 0 for no symptoms and 1 for having symptoms.

The history of respiratory disorders contained 11 questions which were classified into 2 groups: * (1) respiratory irritation symptoms* including (1) coughing in the morning when they wake up; (2) coughing during the daytime or night-time; (3) coughing during the daytime or night-time for >3 months over a period of 1 year; (4) having phlegm regularly on waking; (5) having phlegm frequently during the daytime or night-time; (6) having phlegm for about 3 months over a 1-year period; (7) having a stuffy nose, chest discomfort, or inflammation in the nose when the temperature is low and* (2) allergic symptoms* including (8) difficulty breathing after being exposed to MDF dust; (9) chest discomfort when exposed to MDF dust; (10) being more exhausted than people of the same age when walking on a flat surface; (11) having wheezing during the daytime or night-time, which was divided into two groups of symptoms: irritation and dyspnea.

#### 2.3.2. Data Collection for Exposure to MDF Dust

The MDF dust was sampled using the HSE method MDHS 14/3, which was the general method for sampling and for the gravimetric analysis of respirable and inhalable dusts [29]. The tools used for measurement were the IOM Sampler (US Patent Number 4,675,034) (SKC Inc. Entech Associate Co. Ltd, Bangkok, Thailand), 25 mm PVC filter paper (SKC Inc. Entech Associate Co. Ltd, Bangkok, Thailand), and a personal sampling pump with a flow rate of 2 L/min (SKC Model 224-PCXR8) [30]. The total duration of collection was 8 hours, and the container with the air samples was frozen before being analyzed in the laboratory.

#### 2.3.3. Data Collection for Formaldehyde Exposure

The equipment used to analyze the air samples for formaldehyde exposure was in compliance with the standards set by the US National Institute of Occupational Safety and Health (NIOSH Method 5700; Formaldehyde on Dust) [31]. The equipment used for formaldehyde analysis was high-performance liquid chromatography (HPLC). The analysis was conducted at the Reference Center of the Toxicological Laboratory of the Bureau of Occupational and Environmental Diseases of the Ministry of Public Health, Thailand.

### 2.4. Statistical Analysis

Data analysis was conducted using the statistical software package SPSS (version 22), and the results were presented in tables, frequencies, percentages, means, standard deviations (SD), medians, and minimum and maximum values to explain general variables in the data. Bivariate logistic analysis was used to analyze the association between each independent variable and respiratory symptoms individually. Significant variables including (1) education, (2) atopic eczema, allergic asthma, and allergic rhinitis history, (3) family history, and (4) formaldehyde concentration (ppm) or MDF dust concentration (mg/m^3^) were used to identify the significant variables contributing to dependent variable and the respiratory symptoms in terms of irritation symptoms and allergic symptoms for MDF furniture factory workers by multiple logistic regression analysis.

## 3. Results

### 3.1. Subject Characteristics

The majority of the employees exposed to MDF dust (66.6%) were women. Additional information included the following: their average age was 39.72 years (SD 9.4); 52.6% of the employees had completed primary school; the majority of the employees (80.6%) never smoked, 27.8% were ex-smokers, and only 19.4% of employees were still smoking.

### 3.2. Allergic History

The majority of employees had experience with atopic allergic asthma, allergic rhinitis (25.1%), family allergic history (8%), and metal allergic history (18.51%).

### 3.3. Working History and Work Environment

The majority of employees worked in the packing department (12.50%). Among employees in all departments, those in clearing department (100%) were exposed to formaldehyde at highest level (6.89 ppm). Employees in drilling department (26.3%) were exposed to MDF dust at the highest level (6.37 mg/m^3^). Employees in office (31.1%) were exposed to formaldehyde at the lowest level (0.66–3.44 ppm) as well as 100% of employees being exposed to MDF dust at lowest level (1.71–3.18 mg/m^3^). 41.7% had been working for >10 years (average duration of work 10.7 years; SD 8.14), and 62% wore masks for dust protection (see Table 1).

Results from the analysis of formaldehyde concentration in MDF dust (100 samples) showed that the highest value was 20.85 ppm in the clearing department whereas among the office workers it was 4.21 ppm. The average level was 2.62 ppm (SD 3.67). This was higher than the standard set by OSHA (1987), as shown in Table 2.

The results from the analysis of the concentration of MDF dust (60 samples) showed that the highest level of 13.09 mg/m^3^ was in the fitting department. The average was 7.67 mg/m^3^ (SD 3.63). This was higher than the standard set by ACGIH (2015), as shown in Table 2.

### 3.4. The Health Effects on the Respiratory System

As regards the employees' illness information, there were two symptom groups for respiratory system disorders: irritation and allergic symptoms.

The majority of the employees had symptoms of respiratory system disorders including irritant symptoms and allergic symptoms. For irritant symptoms, 36.4% of the employees who had been exposed to formaldehyde at a highest level of concentration (>6.89 ppm) had coughing during the daytime and night-time and 18.2% had coughing in the morning when they woke up; 35.8% of those who had been exposed to MDF dust at a concentration >6.37 mg/m^3^ also had coughing during the daytime or night-time, and 22.1% had coughing in the morning when they woke up, as shown in Table 3.

For the allergic symptoms, the majority of employees had a history of respiratory system disorders: 18.2% of employees who had been exposed to formaldehyde concentrations >6.89 ppm showed symptoms of having wheeze during the daytime or night-time, as did 18.4% of those who had been exposed to concentrations of MDF dust >6.37 mg/m^3^, as shown in Table 4.

### 3.5. Factors Associating the Respiratory Irritation Symptoms and Allergic Symptoms due to Formaldehyde Exposure

According to this study, employees who had experienced with atopic eczema, allergic asthma, and allergic rhinitis could have an increased risk for irritation symptoms [OR (95% CI) 4.552 (2.350, 8.820)] and allergic symptoms [OR (95% CI) 4.601 (2.375, 8.914)], as shown in Table 5.

### 3.6. Factors Associating Respiratory Irritation Symptoms and Allergic Symptoms due to MDF Dust Exposure

Employees exposed to MDF dust with a history of atopic eczema, allergic asthma, and allergic rhinitis had an increased risk for irritation symptoms [OR (95% CI) 4.293 (2.212, 8.333)] and allergic symptoms [OR (95% CI) 4.340 (2.237, 8.420)]. Exposure to MDF dust at high level (>5 mg/m^3^) was associated with respiratory irritation symptoms and allergic symptoms (aOR; 95% CI: 2.168; 1.380, 3.408 and 2.140; 1.361, 3.364, respectively), as shown in Table 6.

## 4. Discussion

### 4.1. Work History, Allergic History, and Respiratory Symptoms

The employees exposed to formaldehyde at a highest level of concentration (>6.89 ppm) had cough during the day and night, and 18.2% had cough in the morning when they woke up; 35.8% of those who were exposed to MDF dust at a concentration >6.37 mg/m^3^ also had cough during the day and night, and 22.1% had cough in the morning when they woke up. We found that 18.5% of those with metal allergic history were a large proportion and were in agreement with a study by Boonchai et al. [32] in that the most frequent allergen causing allergy among the Thais was potassium dichromate (27%), followed by nickel sulfate (26.6%).

These kinds of sensitizing exposure may cause chronic persistent symptoms and make employees leave the factory. From the interview with the safety officer in this company, we found that the turnover rate of the employees is about 5% monthly. This may be due to the illness caused by chemical exposure in the manufacturing process. From this current study, the majority of the workers had worked for more than 6 years which could probably be the result of the healthy worker effect. Additionally, majority of the workers working for more than 6 years were exposed to a low level of formaldehyde and MDF dust. The survival workers could bear to remain working. The problem of selection bias in this cross-sectional study has been concerned.

We found that the formaldehyde concentration in all departments had an average level of 2.62 ppm (SD 3.67), which was higher than the PELs of OSHA (0.75 ppm) [20]. The concentration was also higher than that found by Kenneth Chung et al. [1], who reported an air concentration of free formaldehyde from MDF of <0.17 mg/m^3^, and was also higher than the result found by Vaizoğlu et al. [21] at 0.6 ppm (SD 0.3). According to this study, the working department that had the highest concentration of formaldehyde was the clearing department, where the tasks required polishing the wood surface and spreading glue on the board. Formaldehyde can be dissipated from resin and glue from the MDF wood into the atmosphere. The working tasks in the furniture making process are different; for example, in the laminating department, the tasks are to place wood onto a conveyor belt, to spread glue, to finish the wood surface. In the packing department the tasks include packing the wood into a package made from a paper box, including gluing parts in order to make the box and then packing the wood into the box. In the combination department, the task is to cut the wood into particular shapes. The three departments showed a mean formaldehyde concentration of 4.55 ppm (SD 3.09), 1.89 ppm (SD 2.28), and 0.90 ppm (SD 1.85), respectively, whereas the concentration in the office was 1.52 ppm (SD 1.48). It was also higher than the standard set by OSHA [20].

According to Tables 1 and 2 fewer workers were exposed to moderate or high concentration of formaldehyde and MDF dust. However, it is surprising that the concentration of formaldehyde in the office was also higher than that of OSHA standard [20] because the office was located in the same area as the production. Office workers and customers walked in and out of the office all day. Formaldehyde which is in the gas phase may be spread from the sources into the office and throughout the factory. The main threat to employees in the furniture making process is most likely from the fugitive formaldehyde emissions in the form of gas released from MDF materials and finished products.

### 4.2. Respiratory Irritation Symptoms and Formaldehyde Exposure

Employees with atopic allergic history and family allergic history had an increased risk of respiratory irritation symptoms. Formaldehyde concentration was associated with respiratory irritation symptoms. This finding may be due to the healthy worker effect. However, due to the cross-sectional design of the study, respiratory symptoms might appear before the study was conducted. Thus, the reverse association could be found. The result of the study may support the association between high (exceeding TLV) concentration of formaldehyde and respiratory symptoms.

Inhalation of formaldehyde can cause irritation in the respiratory system [33–35] because formaldehyde can be absorbed into the mucus of the nose and throat, as well as the eyes. Inhaling different concentrations of formaldehyde can also have different effects on the body. However, if employees were exposed to a concentration of 5 ppm, which is close to our result of 2.78 ppm (SD 3.85), it would cause lower respiratory tract irritation. Similar to the study of Monticello et al. [35], the symptoms of this irritation included cough, chest tightness, and/or wheezing. This result agrees with those of a study conducted in animals showing that formaldehyde caused sensory irritation at concentrations >1 ppm [34], as well as with a study in humans conducted in a laboratory with the participation of healthy subjects. The sample group was exposed to formaldehyde at the concentration of 2.0 ppm, and the results showed symptoms of irritation [36]. Another study looked at 21 samples that were exposed to formaldehyde at concentrations of 0.12 and 1.6 ppm, as well as 18 samples from a nonexposed group, and the results again showed symptoms of eye, nose, and throat irritation in the exposed group [37].

### 4.3. Respiratory Allergic Symptoms and Formaldehyde Exposure

We found that employees who had atopic allergic history and family allergic history had an increased risk of respiratory allergic symptoms. However, this is possibly the consequence of off the job exposure which was not included in the analysis. The current study found that employees who had a history of atopic dermatitis, asthma, and allergic rhinitis were more likely to develop similar symptoms. In addition, persons with a level of high sensitivity would experience reactions to their environment more quickly. This current study found similar results as in the study of Mortz et al. [38], who indicated that persistent atopic dermatitis was particularly prevalent in those with early-onset allergic rhinitis and hand eczema in childhood. Bell and King [9] also found that male workers with atopic eczema whose jobs involved cutting MDF laminated flooring had a positive patch test for urea–formaldehyde resin.

Formaldehyde is a substance that can cause asthma [6, 39]. OSHA [20] has stated that formaldehyde is a stimulant that causes pulmonary sensitization, which is a cause of lung disorders. An experiment on rats [40] showed that exposure to formaldehyde resulted in bronchial constriction. It is possible that, after being inhaled, formaldehyde-laden dust could pass into the lower respiratory tract, which might lead to respiratory system disorders and the impairment of lung function. Therefore, health monitoring is necessary for employees exposed to formaldehyde. Exposure to MDF dust can also have long-term effects on lung function [7].

The results of this study correspond to those of a previous study involving 15 workers who were exposed to formaldehyde, showing that the exposed group had developed asthma, possibly resulting from hypersensitivity to this substance [14, 41]. They also correspond to the study conducted by Burton et al. [7], which indicated that shortness of breath and chest tightness could develop after exposure to glues, resin, polyvinyl acetate, and urea–formaldehyde resin-based wood glue from the MDF woodworking process.

Inhalation of formaldehyde vapor could lead to respiratory symptoms such as irritation, asthma, and allergies. Recent studies on asthma and airway biology have implicated changes in the disposition of nitric oxide (NO) in the adverse effects of formaldehyde, which can lead to bronchoconstriction [42]. Exposure to dusts containing formaldehyde resin is related to obstructive lung diseases such as bronchitis, and asthmatic reactions can develop after exposure to formaldehyde resin dust and gas. The physical and chemical properties of formaldehyde might be the cause of asthma [39], which corresponds to a study [37] on subjects exposed to formaldehyde at concentrations of 0.12 and 1.6 ppm. The symptoms found in the exposed group included chest tightness and shortness of breath, although lung function did not deteriorate.

Our results concerning allergic respiratory symptoms due to exposure to formaldehyde showed that a relationship was found between allergic symptoms results and educational level. Primary and elementary educational levels had higher risk of allergic respiratory symptoms than university education level. This current study was similar to the study of Gonzalez-Garcia et al. [43], indicating that educational level has a significant relationship with respiratory symptoms. However, the level of formaldehyde concentrations was too high for all workers working in this factory. Health education among atopic or allergic workers may not show any benefit.

Additionally, the current study found that workers who were at-risk of exposure to formaldehyde included those who had the histories of atopic eczema, allergic asthma, allergic rhinitis, family allergic history, and low education. Nonetheless, excluding allergic workers from study population produced same kind of results in analysis as among total population; that is, those symptoms of all workers are associated with high concentrations of air impurities in this factory. Low education is associated with higher exposure and higher prevalence of respiratory symptoms. It might be a confounder, and not really the result.

This group of employees should be regularly monitored as part of the health surveillance program. It is necessary to consider controlling exposure to other factors that may cause respiratory problems among the workers in this factory. In addition, it is essential to pay attention to controlling the concentration of formaldehyde in the work environment despite the regulation on formaldehyde standard in Thailand, and such standards should be promulgated.

### 4.4. Respiratory Irritation Symptoms and MDF Dust Exposure

According to this study, the area where the mean concentration of MDF dust was lowest in the office area, at 1.71 mg/m^3^, whereas the combination department had the highest concentration of 11.24 mg/m^3^. The mean level in all departments was 7.67 mg/m^3^ (SD 6.49), which was higher than the standard set by ACGIH [22], which states that wood dust is a human carcinogen and suggests that exposure should not exceed 1 mg/m^3^ for hardwood and 5 mg/m^3^ for softwood.

The factors that contributed to respiratory irritation occurred when the concentration of inhalable MDF dust at >5 mg/m^3^ was (aOR (95% CI) 2.1681; 1.380, 3.408). Employees with atopic history had an increased risk of respiratory allergic symptoms (aOR (95% CI) 4.340; 2.237,8.420). This current study was similar to the study of Mortz et al. [38], who indicated that persistent atopic history was particularly prevalent in those with early-onset allergic rhinitis in childhood. The properties of MDF dust are different from those of natural wood. There are four types of wood dust in the aspects of dust production: MDF, urea–formaldehyde resin, natural softwood (pine), and natural hardwood (oak). The sanding process for MDF produces more dust than natural woods (hard or soft), but sawing produces the same amount [1]. The study also found that MDF dust led to more effects on the upper airway than dust from natural wood [8]. When excluding allergic employees from the analysis, the result indicated that a significant association between MDF dust concentration and irritation symptoms was found. This evidence supported that MDF dust could cause irritation symptoms.

### 4.5. Respiratory Allergic Symptoms and MDF Dust Exposure

In addition, MDF dust concentration of >5 mg/m^3^ was also related to allergic respiratory symptoms (aOR (95% CI) 2.140; 1.361, 3.364). Our findings correspond to those of study on work-related allergic conditions due to MDF, which indicated that workers had developed symptoms of occupational rhinitis. This study suggested that the symptoms resulted from exposure to formaldehyde combined with exposure to wood dust, leading to nasal dysfunction and irritation to the nasal mucosa. It is possible that fine particles of dust released from the board had absorbed formaldehyde, leading to allergic symptoms in the group who had been exposed to the dust [44, 45].

Specially when excluding allergic employees from the analysis, a significant association between MDF dust concentration and allergic respiratory symptoms was found. MDF dust exposure might be the cause of allergic symptoms.

### 4.6. Limitations of the Study

The limitations of this current study are that the symptom complaints were discovered from the questionnaire answered by the subjects, not from a medical check by a physician. Also, the lung function tests for exposure to formaldehyde and MDF dust were not assessed in the at-risk group. Respiratory symptoms were subjective and very limited. Further studies should provide information that can proof the effects of the respiratory system caused by MDF dust and formaldehyde such as a biomarker. Additionally, off the job exposure was not included in the analysis.

## 5. Conclusions 

This study found the evidence that formaldehyde concentration was 3.5 times above the standard concentration set by the Occupational Safety and Health Administration (OSHA). Regarding exposure to MDF dust, MDF dust concentration >5 mg/m^3^ was related to irritation and allergic symptoms. Therefore, it is advisable that controls of exposure to MDF dust should be imposed in accordance with the law; however, Thailand still has no regulations or standards for concentrations of inhalable dust in the workplace, and such standards should therefore be formulated. In addition, it is necessary to pay attention to the control of respiratory exposure in the production processes of this factory.

## Figures and Tables

**Table 1:** Work history.

Variables	Formaldehyde exposed group (ppm)			Total *N* = 439 (%)	MDF dust exposed group (mg/m^3^)			Total *N* = 439 (%)
	Low (0.66–3.44)	Moderate (3.45–6.89)	High (>6.89)		Low 1.71–3.18	Moderate (3.19–6.37)	High (>6.37)	
*Department*								
Office	116 (31.1)	0 (0)	0 (0)	116 (26.4)	116 (100)	0 (0)	0 (0)	116 (26.4)
Combination	39 (10.5)	0 (0)	0 (0)	39 (8.9)	0 (0)	0 (0)	39 (20.5)	39 (8.9)
Edging	51 (13.7)	0 (0)	0 (0)	51 (11.6)	0 (0)	51 (38.3)	0 (0)	51 (11.6)
Fitting	34 (9.1)	0 (0)	0 (0)	34 (7.4)	0 (0)	0 (0)	34 (17.9)	34 (7.4)
Clearing	0 (0)	0 (0)	22 (100)	22 (5)	0 (0)	0 (0)	22 (11.6)	22 (5)
Drilling	50 (13.4)	0 (0)	0 (0)	50 (11.41)	0 (0)	0 (0)	50 (26.3)	50 (11.41)
Laminating	0 (0)	27 (61.4)	0 (0)	27 (6.2)	0 (0)	27 (20.3)	0 (0)	27 (6.2)
Wrapping	28 (7.5)	0 (0)	0 (0)	28 (6.4)	0 (0)	0 (0)	28 (14.7)	28 (6.4)
Packing	55 (14.7)	0 (0)	0 (0)	55 (12.5)	0 (0)	55 (41.4)	0 (0)	55 (12.5)
Line	0 (0)	17 (38.6)	0 (0)	17 (3.9)	0 (0)	0 (0)	17 (8.9)	116 (26.4)
*Work duration (yrs)*								
<1	48 (12.9)	9 (20.9)	2 (9.1)	59 (13.5)	10 (8.6)	22 (16.7)	27 (14.4)	59 (13.5)
1-2	14 (3.8)	1 (2.3)	0 (0)	15 (3.4)	6 (5.2)	4 (3)	5 (2.7)	15 (3.4)
3–5	48 (12.9)	0 (14)	2 (9.1)	56 (12.8)	18 (5.5)	17 (12.9)	2 (11.2)	56 (12.8)
6–10	11 (25.6)	4 (18.2)	4 (18.2)	124 (28.4)	57 (31.9)	27 (28)	50 (26.6)	124 (28.4)
>10	16 (37.2)	16 (37.2)	14 (63.6)	182 (41.7)	45 (38.8)	52 (39.4)	85 (45.2)	182 (41.7)
*Overtime work (hour/week)*								
1–6	267 (71.6)	40 (90.9)	22 (100)	329 (74.9)	107 (92.2)	95 (71.4)	127 (66.8)	329 (75.9)
>6	106 (28.4)	4 (9.1)	0 (0)	110 (25.1)	9 (7.8)	38 (28.6)	127 (66.8)	110 (25.1)
*PPE use*	215 (57.6)	37 (84.1)	20 (90.9)	272 (62)	27 (23.3)	100 (75.2)	145 (76.3)	272 (62.0)

**Table 2:** Level of formaldehyde concentration (ppm) and MDF wood dust (mg/m^3^) in the work environment.

Department	*N* = 100	Formaldehyde concentration (ppm)	Department	*N* = 60	MDF wood dust concentration (mg/m^3^)
*Office*	12		*Office*	6	
Mean (SD)		1.52 (1.48)	Mean (SD)		1.71 (0.98)
Median (min, max)		1.09 (0.0, 4.21)	Median (min, max)		1.69 (0.66, 3.17)
*Combination*	10		*Combination*	6	
Mean (SD)		0.90 (1.85)	Mean (SD)		11.24 (0.40)
Median (min, max)		0 (0, 5.37)	Median (min, max)		11.38 (10.74, 11.59)
*Edging*	10		*Edging*	6	
Mean (SD)		0.68 (1.44)	Mean (SD)		5.62 (0.41)
Median (min, max)		0.0 (0.0, 4.51)	Median (min, max)		5.65 (4.88, 6.14)
*Fitting*	11		*Fitting*	6	
Mean (SD)		2.36 (2.01)	Mean (SD)		6.54 (3.23)
Median (min, max)		2.29 (0.0, 5.97)	Median (min, max)		5.40 (4.46, 13.09)
*Clearing*	9		*Clearing*	6	
Mean (SD)		8.32 (7.22)	Mean (SD)		10.46 (1.24)
Median (min, max)		5.03 (0.43, 20.85)	Median (min, max)		10.51 (8.92, 11.97)
*Drilling*	8		*Drilling*	6	
Mean (SD)		0.57 (0.65)	Mean (SD)		11.10 (1.23)
Median (min, max)		0.37 (0.0, 1.70)	Median (min, max)		11.72 (9.06, 12.17)
*Laminating*	10		*Laminating*	6	
Mean (SD)		4.55 (3.09)	Mean (SD)		5.99 (1.60)
Median (min, max)		4.32 (0.39, 11.96)	Median (min, max)		5.64 (4.49, 9.02)
*Wrapping *	10		*Wrapping *	6	
Mean (SD)		1.41 (1.76)	Mean (SD)		10.04 (2.74)
Median (min, max)		0.59 (0.0, 4.12)	Median (min, max)		11.71 (6.37, 11.92)
*Packing*	10		*Packing*	6	
Mean (SD)		1.89 (2.28)	Mean (SD)		3.96 (1.24)
Median (min, max)		0.98 (0.0, 6.57)	Median (min, max)		3.21 (3.07, 5.69)
*Line*	10		*Line*	6	
Mean (SD)		4.46 (3.91)	Mean (SD)		10.04 (2.74)
Median (min, max)		4.07 (0.0, 14.0)	Median (min, max)		11.71 (6.37, 11.92)
*Total*	100		*Total*	60	
Mean (SD)		2.62 (3.67)	Mean (SD)		7.67 (3.63)
Median (min, max)		1.32 (0.0, 20.85)	Median (min, max)		6.50 (0.66, 13.09)

**Table 3:** Respiratory irritation symptoms.

Variables	Formaldehyde exposed Group (ppm)			Total *N* = 439 (%)	MDF dust exposed group (mg/m^3^)			Total *N* = 439 (%)
	Low (0.66–3.44)	Moderate (3.45–6.89)	High (>6.89)		Low (1.71–3.18)	Moderate (3.19–6.37)	High (>6.37)	
(1) Cough in the morning when they wake up	67 (18)	11 (25)	4 (18.2)	82 (18.7)	17 (14.7)	23 (17.3)	42 (22.1)	82 (18.7)
(2) Cough during the daytime or night-time	103 (27.6)	14 (31.8)	8 (36.4)	125 (28.5)	21 (18.1)	36 (27.1)	68 (35.8)	125 (28.5)
(3) Cough during the daytime or night-time for >3 months over a period of 1 year	40 (10.7)	6 (6.8)	1 (4.5)	44 (10)	7 (6)	13 (9.8)	24 (12.6)	44 (10)
(4) Having phlegm regularly on waking	63 (16.9)	6 (13.6)	3 (13.6)	72 (16.4)	11 (9.5)	20 (15.0)	41 (21.6)	72 (16.4)
(5) Having phlegm frequently during the daytime or night-time	44 (11.8)	6 (13.6)	1 (4.5)	51 (11.6)	10 (8.6)	9 (6.8)	32 (16.8)	51 (11.6)
(6) Having phlegm for about 3 months over a 1-year period	58 (15.5)	8 (18.2)	0 (0)	66 (15)	12 (10.3)	16 (12)	38 (20)	66 (15)
(7) Having a stuffy nose, chest discomfort, or inflammation in the nose when the temperature is low	104 (27.9)	8 (18.2)	9 (40.9)	121 (27.6)	21 (18.1)	34 (25.6)	66 (34.7)	121 (27.6)

**Table 4:** Respiratory allergic symptom.

Variables	Formaldehyde exposed group (ppm)			Total *N* = 439 (%)	MDF dust exposed group (mg/m^3^)			Total *N* = 439 (%)
	Low (0.66–3.44)	Moderate (3.45–6.89)	High (>6.89)		Low (1.71–3.18)	Moderate (3.19–6.37)	High (>6.37)	
(1) Difficulty breathing after being exposed to MDF dust	27 (7.2)	3 (6.8)	0 (0)	30 (6.8)	5 (4.3)	7 (5.3)	18 (9.5)	30 (6.8)
(2) Chest discomfort when exposed to MDF dust	90 (24.2)	6 (13.6)	3 (13.6)	99 (22.6)	15 (12.9)	33 (25)	51 (26.8)	99 (22.6)
(3) Being more exhausted than people of the same age when walking on a flat surface	54 (14.5)	6 (13.6)	3 (13.6)	63 (14.4)	14 (12.1)	16 (12.0)	33 (17.4)	63 (14.4)
(4) Having wheeze during the daytime or night-time	56 (15)	2 (4.5)	4 (18.2)	62 (14.1)	14 (12.1)	13 (9.8)	35 (18.4)	62 (14.1)

**Table 5:** Factors associating irritation and allergy symptoms among workers exposed to formaldehyde (ppm).

Variables	Irritation				Allergy			
	Crude		Adjusted OP OR 95% (CI)		Crude		Adjusted 95% CI (lower, upper)	
	OR 95% (CI)	*P* value			OR 95% (CI)	*P* value		
*Education*								
Primary school	3.354 (1.648, 6.827)	0.001	3.238	(1.541, 6.801)	3.284 (1.614, 6.684)	0.001	3.615	(1.506, 6.652)
Elementary school	3.209 (1.581, 6.512)	0.001	2.853	(1.359, 6.987)	3.209 (1.581, 6.512)	0.001	2.852	(1.358, 5.988)
≥University	Ref	Ref	Ref	Ref	Ref	Ref	Ref	Ref
*Atopic eczema, allergic rhinitis history*								
No	Ref	Ref	Ref	Ref	Ref	Ref	Ref	Ref
Yes	4.77 (2.499, 9.091)	0.000	4.552	(2.350, 8.820)	4.818 (2.526, 9.189)	0.000	4.601	(2.375, 8.914)
*Family allergic history*								
No	Ref	Ref	Ref	Ref	Ref	Ref	Ref	Ref
Yes	2.872 (1.345, 6.135)	0.006	2.686	(1.294, 6.358	2.901 (1.358, 6.197)	0.006	2.898	(1.307, 6.425)
*Formaldehyde (ppm)*								
<0.75	Ref	Ref	Ref	Ref	Ref	Ref	Ref	Ref
≥0.75	0.362 (1.581, 6.512)	0.002	0.365	(0.188, 0.707)	0.358 (1.89, 0.677)	0.007	0.361	(0.186, 0.700)

*Note: the respiratory irritation symptoms:* (1) coughing in the morning when they wake up; (2) coughing during the daytime or night-time; (3) coughing during the daytime or night-time for >3 months over a period of 1 year; (4) having phlegm regularly on waking; (5) having phlegm frequently during the daytime or night-time; (6) having phlegm for about 3 months over a 1-year period; and (7) having a stuffy nose, chest discomfort, or inflammation in the nose when the temperature is low. *The allergic symptom:* (1) difficulty breathing after being exposed to MDF dust; (2) chest discomfort when exposed to MDF dust; (3) being more exhausted than people of the same age when walking on a flat surface; (4) having wheezing during the daytime or night-time.

**Table 6:** Factors associating irritation and allergy symptoms among workers exposed to MDF wood dust (mg/m^3^).

Variables	Irritation				Allergy			
	Crude		Adjusted OP OR 95% (CI)		Crude		Adjusted 95% CI (lower, upper)	
	OR 95% (CI)	*P* value			OR 95% (CI)	*P* value		
*Education*								
Primary school	3.354 (1.648, 6.827)	0.001	1.994	(0.893, 4.448)	3.284 (1.614, 6.684)	0.001	1.967	(0.881, 4.392)
Elementary school	3.209 (1.581, 6.512)	0.001	1.986	(0.906, 4.351)	3.209 (1.581, 6.512)	0.001	2.002	(0.914, 4.388)
≥bachelor	Ref	Ref	Ref	Ref	Ref	Ref	Ref	Ref
*Atopic eczema, allergic rhinitis history*								
No	Ref	Ref	Ref	Ref	Ref	Ref	Ref	Ref
Yes	4.77 (2.499, 9.091)	0.000	4.293	(2.212, 8.333)	4.818 (2.526, 9.189)	0.000	4.340	(2.237, 8.420)
*Family allergic history*								
No	Ref	Ref	Ref	Ref	Ref	Ref	Ref	Ref
Yes	2.872 (1.345, 6.135)	0.006	3.005	(1.347, 6.702)	2.901 (1.358, 6.197)	0.006	3.029	(1.358, 6.756)
*MDF dust (mg/m* ^*3*^)								
≤5	Ref	Ref	Ref	Ref	Ref	Ref	Ref	Ref
>5	0.362 (1.581, 6.512)	0.002	2.168	(1.380, 3.408)	257 (1.697, 3.758)	0.000	2.140	(1.361, 3.364)

*Note: the respiratory irritation symptoms:* (1) coughing in the morning when they wake up; (2) coughing during the daytime or night-time; (3) coughing during the daytime or night-time for > 3 months over a period of 1 year; (4) having phlegm regularly on waking; (5) having phlegm frequently during the daytime or night-time; (6) having phlegm for about 3 months over a 1-year period; and (7) having a stuffy nose, chest discomfort, or inflammation in the nose when the temperature is low. *The allergic symptoms:* (1) difficulty breathing after being exposed to MDF dust; (2) chest discomfort when exposed to MDF dust; (3) being more exhausted than people of the same age when walking on a flat surface; (4) having wheezing during the daytime or night-time.
